# Intensification of extraction process through IVDV pretreatment from *Eryngium creticum* leaves and stems: Maximizing yields and assessing biological activities

**DOI:** 10.1016/j.heliyon.2024.e27431

**Published:** 2024-03-08

**Authors:** Mariam Hammoud, Espérance Debs, Lambertus A.M. van den Broek, Hiba N. Rajha, Carl Safi, Gijs van Erven, Richard G. Maroun, Ali Chokr, Hassan Rammal, Nicolas Louka

**Affiliations:** a Centre d’Analyses et de Recherche, Unité de Recherche Technologies et Valorisation Agro-alimentaire, Faculté des Sciences, Université Saint-Joseph, B.P. 17-5208 Riad El Solh, Beirut, Lebanon; b Research Laboratory of Microbiology (RLM), Department of Life and Earth Sciences, Faculty of Sciences I, Lebanese University, Hadath Campus, Beirut, Lebanon; c Platform of Research and Analysis in Environmental Sciences (PRASE), Doctoral School of Sciences and Technology, Lebanese University, Hadath Campus, Beirut, Lebanon; d Department of Biology, Faculty of Arts and Sciences, University of Balamand, Tripoli P. O. Box 100, Lebanon; e Wageningen Food & Biobased Research, PO Box 17, 6700 AA Wageningen, the Netherlands; f Ecole Supérieure d’Ingénieurs de Beyrouth (ESIB), Saint-Joseph University, Mkalles Mar Roukos, Beirut, Lebanon; g Faculty of Agronomy, Lebanese University, Dekweneh-Lebanon

**Keywords:** *E. creticum*, IVDV, Optimization, Phenolic compounds, Response surface methodology, Antibacterial and antibiofilm activities, Stereomicroscopy

## Abstract

“Intensification of Vaporization by Decompression to the Vacuum” (IVDV) has initially emerged as a technology primarily employed for expanding and enhancing the texture of biological products. However, its recent applications have showcased significant promise in the realm of extracting bioactive molecules from various plant materials. In this context, optimization using response surface methodology was conducted to investigate the impact of IVDV pretreatment on the extractability of phenolic compounds from *Eryngium creticum* leaves and stems, as well as their biological activities. Using IVDV preceding the extraction led to higher total phenolic content (TPC) and enhanced antiradical activities in treated materials compared to untreated ones. The optimal processing conditions in terms of water content, steam pressure and treatment time were determined in order to maximize TPC (89.07 and 20.06 mg GAE/g DM in leaves and stems, respectively) and antiradical (DPPH) inhibition percentage (93.51% and 27.54% in leaves and stems, respectively). IVDV-treated extracts showed superior antioxidant, antibacterial and antibiofilm capacities compared to raw extracts. Using RP-UHPLC-PDA-MS, caffeic acid and rosmarinic acid were identified in IVDV-treated leaves. IVDV can be implemented as an innovative treatment applied prior to extraction to boost the recovery of biomolecules from plant matrices.

## Highlights

• IVDV pretreatment intensifies polyphenols' recovery from *E. creticum* plant.
• Leaves extracts exhibited higher antioxidant, antibacterial and antibiofilm capacities compared to stems extracts.
• IVDV pretreatment induces product's alveolation due to the drop in saturated steam pressure towards vacuum.
• Caffeic acid and rosmarinic acid (derivatives) were analyzed by RP-UHPLC-PDA-MS in IVDV-treated leaves extract.

## 1 Introduction

The biological properties of medicinal plants have traditionally been associated with the presence of bioactive molecules, which are specific chemical compounds responsible for the observed therapeutic potential. These bioactive molecules encompass a wide range of compounds, among others alkaloids, flavonoids, terpenoids, and phenolic compounds. Extensive scientific research has been conducted to elucidate their specific applications in healthcare. *Eryngium creticum* L., is an edible plant referred to as “snakeroot,” found in the Middle East region, primarily recognized for its use as a remedy for scorpion and snake bites [1]. It is a spiny herbaceous plant extending at a height of 50 cm, featuring a straight branched stem [2]. Traditionally, *E. creticum* has been used to address various health issues, many of which have been corroborated by scientific investigations. These studies demonstrated the plant's diverse biological activities, including properties against snake and scorpion venom, and in addition antitumor, antimicrobial, antioxidant, and hypoglycemic activities [[3], [4], [5], [6], [7], [8]].

The extraction process employed crucially affects the retrieval of bioactive molecules from plant materials [9,10]. The efficacy of an extraction technique is not solely determined by its ability to boost the final yield of biomolecules; it must also minimize extraction duration and organic solvent consumption while ensuring the quality of the target molecules [11]. In addition to the water bath extraction (conventional method), several innovative extraction techniques have been explored by our team in order to enhance the recovery yield, including ultrasound, voltage electrical discharges, infrared irradiation, pulsed electric field, and others [[12], [13], [14], [15], [16], [17]].

Besides, other technologies, exerting a thermo-mechanical effect, can be applied to further improve the extraction, such as explosion puffing. Traditionally used for enhancing drying, explosion puffing has been adapted for biomolecule extraction [18]. This method involves injecting saturated steam under high pressure for a given treatment time. At the end, the pressure in the treatment vessel is decompressed back to the atmospheric level inducing a porous structure in the puffed material. This porosity facilitates the release of internal bioactive compounds during subsequent extraction. Another expansion process, known as “Détente Instantanée Contrôlée” (DIC), differs from the traditional puffing due to a swift decompression to the vacuum (10^4^ Pa) instead of the atmospheric pressure [19,20]. It was employed to further modify the structure of the treated products. Recently, the “Intensification of Vaporization by Decompression to the Vacuum” (IVDV) technology was developed to be even more efficient. This improvement includes a very rapid pressurization system, allowing a high-pressure level to be reached within less than 1 s, compared to around 10 s required by similar technologies. The sudden pressure drop to the vacuum within a very brief period is crucial for treating thermo-sensitive products that cannot withstand prolonged exposure to high pressure/temperature. IVDV is an emergent process that was initially designed as an expansion technique applied in texturizing fruits, vegetables, and other biological products [21,22], while preserving their nutritional content and ameliorating their sensory properties [23].

In the context of this study, IVDV technique demonstrated noteworthy potential in enhancing the extraction yield from plant materials [24]. Our aim was to explore the application of IVDV as a pretreatment on *E. creticum* leaves and stems, prior to conventional water bath extraction, to intensify the recovery of materials of interest from the original matrix. Response Surface Methodology (RSM) was applied to maximize the recovery yield while preserving the antiradical activity of the extract. Antioxidant and antibacterial capacities of the extracts obtained from IVDV-treated leaves and stems were subsequently assessed, and compared to raw extracts. Finally, the phenolic profile of IVDV-treated leaves extract was tentatively identified using RP-UHPLC-PDA-MS analysis.

## 2 Materials and methods

### 2.1 Chemicals, media, and bacterial strains

Folin-Ciocalteu reagent, 2,2-diphenyl-picrylhydrazyl (DPPH), sodium carbonate, gallic acid, Trolox, as well as all extraction solvents, were of analytical grade and acquired from Sigma-Aldrich (Steinheim, Germany).

Mueller Hinton Broth (MHB), Tryptic Soy Broth (TSB) and Brain Heart Agar (BHA) were obtained from HIMEDIA (Mumbai, India). Bacteria used in this study included *Staphylococcus aureus* (ATCC 49619) and *Staphylococcus epidermidis* RP62A (ATCC 35984), both Gram-positive strains, and *Escherichia coli* (ATCC 35218) and *Pseudomonas aeruginosa* (ATCC 27853), both Gram-negative strains. Before use, these bacteria were stored in glycerol at −80 °C.

### 2.2 Plant material

The plants were harvested from Lebanon Southern governorate (400-m altitude) (33.3901° N, 35.4183° E) and identified according to a certified Lebanese flora reference [25]. After collection, they were placed in an airflow oven for drying for 48 h at 35 °C (UFE 700, Memmert GmbH, Schwabach, Germany). After that, whole leaves and stems were stored protected from sunlight at room temperature before further use.

### 2.3 Dry matter

The dry matter (DM) content of leaves and stems was obtained through heating at 105 °C for 24 h in a ventilated oven [26]. The dry matter content for leaves and stems were 88% (*w*/*w*) and 93% (*w*/*w*), respectively.

### 2.4 Sample preparation and IVDV pretreatment

Sample rehydration is a step that precedes IVDV pretreatment. It was carried out by spraying distilled water on the dried leaves and stems. The hydrated plant materials were stored at 4 °C for 3 days to guarantee homogeneous redistribution and optimal water absorption before subsequent treatment.

**Fig. 1:**
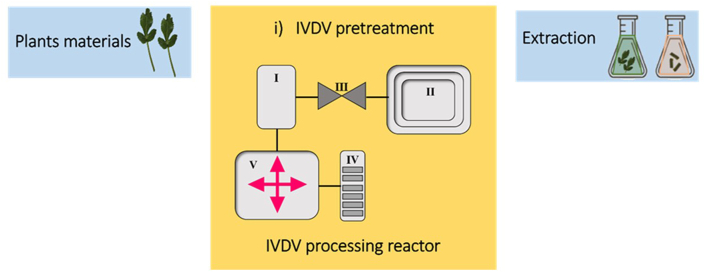
Diagram of the IVDV processing apparatus. (I): processing vessel; (II): vacuum tank; (III): decompression valve; (IV): ultra-speed pressure-increase system; (V): steam generator.

The IVDV processing reactor, as illustrated in Fig. 1, consists of five major parts: the processing vessel (I) in which samples are treated under high saturated steam pressure; a tank (II) in which the vacuum is continuously maintained thanks to a vacuum pump; a six-inches ball valve (III) that connects the processing chamber to the vacuum tank; the ultra-speed pressure-increase system that ensures a fast injection of the saturated steam into the processing vessel (IV) and the steam generator (V).

Samples placed into the processing vessel undergo treatment under high temperature/high pressure for a specific treatment time. The duration of steaming, the applied temperature, as well as the sample's water content are set by the experimental design (paragraph 2.6). The treatment is ended by a sudden pressure drop towards the vacuum (∼0.003 MPa) causing water vaporization water inside the treated sample, thus leading to its alveolation. Rapid cooling was simultaneously applied to prevent further thermal degradation of the product. The originality of this approach lies in the swift increase in steam pressure (up to 12 bar/s) enabled by the ultra-speed pressure-increase system. The final porosity level of the treated materials and the ensuing intensification of mass transfer phenomena (solvent transfer into the solid matrix, and target molecules diffusion from the solid matrix to the surrounding solvent) depend on the operating factors: W “the water content”, P “the steam pressure”, and t “the processing time”.

### 2.5 Extraction procedure

The phenolic compounds extraction was conducted by mixing 1 g of *E. creticum* leaves or stems (control or IVDV-treated) with 50 mL of solvent (ethanol 60%) in an Erlenmeyer flask placed into a water bath. The choice of solvent was determined based on a prior study [27] carried out on similar material. All experiments were performed at 60 °C for a 2-h duration.

### 2.6 Experimental design

Response Surface Methodology was carried out for optimization of the IVDV procedure. A central composite (2^3^ + star) rotatable design, comprising 18 runs with eight factorial design points, six star points, and four central repetitions, was created to assess the correlation between the operating parameters. The studied parameters were W “water content”, P “steam pressure”, and t “processing time”. W varied from 17% to 27%, P from 2.5 bars to 5 bars, and t ranged between 7 s and 17 s for leaves, while W varied from 14.3% to 34.3%, P from 3 bars to 6 bars, and t ranged between 10 s and 20 s for stems. This design was used to evaluate the impact of the operating parameters on Total Phenolic Content (TPC) and antiradical activity (DPPH inhibition percentage) as response variables. The experimental design and statistical analysis were conducted using STATGRAPHICS Centurion XVI.I (Statigraphics 18, The Plains, Virginia).

### 2.7 Total phenolic content (TPC)

The total phenolic content in *E. creticum* leaves and stem extracts was assessed by the Folin-Ciocalteu method [28] as follows: the extract (200 μL) was mixed with ten-fold (1/10 *v/v*) diluted Folin-Ciocalteu reagent (1000 μL) and 800 μL of Na_2_CO_3_ (7.5% *w/v*). The absorbance was measured at 750 nm after incubation of 10 min at 60 °C followed by 10 min at 4 °C. The phenolic content was quantified as milligrams of gallic acid (using as standard) equivalent (GAE) per gram of dry matter (mg GAE/g DM).

### 2.8 Antiradical activity

The measurement of free radical scavenging activity was assessed by the capacity of the phenolic compounds to reduce DPPH [29]. *E. creticum* extracts (50 μL) or positive control (Trolox) were mixed with 1.45 mL of DPPH solution. The absorbance (515 nm) was measured after incubation in the dark at room temperature for 30 min. The inhibition percentage (Inh.%) of the free radical DPPH was calculated as shown in equation (1):

(1) $$Inh.\;\%=\frac{Absorbance\;of\;negative\;control-Absorbance\;of\;sample}{Absorbance\;of\;negative\;control}*100$$

### 2.9 Evaluation of the antioxidant activities

CUPRAC assay (Cupric ion reducing antioxidant capability), FRAP assay (Ferric reducing antioxidant power), and ABTS assay (2,2′-azino-bis-(3-ethylbenzothiazoline-6-sulphonic acid)) were used to measure the capacity of the extracts to oxidate copper II-neocuproine (2,9-dimethyl-1,10-phenanthroline) (expressed as mM Trolox Equivalent (TE mM)), to reduce the ferric complex (expressed as μM of Iron (II) equivalent (μM Iron (II)), and to determine the ABTS scavenging activity (expressed in mM of Ascorbic acid Equivalent (mM AA)), respectively (Bioquochem, Asturias, Spain). Methods used were performed according to Hammoud et al. [27].

### 2.10 Antibacterial assay: minimal inhibitory concentration (MIC) and minimal bactericidal concentration (MBC)

The plant's extracts were evaluated for determination of their corresponding MIC and MBC as suggested by the Clinical and Laboratory Standards Institute using the microdilution method [30]. The procedure followed was same as described by Hammoud et al. [27*,*31]. A serial two-fold dilution of each extract was performed in media (MHB). To reach a final concentration of 5 × 10^5^ CFU/mL, a bacterial inoculum was added, and plates were incubated at 37 °C for 24 h. MIC and MBC were subsequently determined.

### 2.11 Antibiofilm activities

#### 2.11.1 Biofilm formation

The biofilm formation assay was based on a standard procedure [32,33]. Five μL bacterial inoculum was added to each well of a microplate to reach a final concentration of 5 × 10^5^ CFU/mL with the exception of column 12 (negative control). Subsequently, the plates were incubated for 24 h at 37 °C. Afterwards, the contents of wells were discarded and the formed biofilms were heat-fixed for 1 h at 80 °C. Crystal violet was used for staining. To remove any non-adherent bacteria, the microplates were washed with distilled water. Optical density (OD _570 nm_) was measured by an ELISA microplate reader (BioTek Instruments, ELx800, USA). The used procedure was same as described by Hammoud et al. [27,31].

#### 2.11.2 Biofilm eradication activity

Upon fixation of the biofilm, as described above, a serial two-fold dilutions of extracts in sterile distilled water were added in all wells excluding for column 11 (positive control). The microplates were incubated for 18 h at 37 °C. Staining and measurement of the optical density were performed as described by Hammoud et al. [27,31].

#### 2.11.3 Biofilm prevention activity

To evaluate the prevention biofilm activity of extracts, all wells were filled with TSB (100 μL) supplemented with 0.25% glucose (*w/v*). A hundred μL extract was added and a two-fold serial dilution was done. To each well a 5 μL bacterial inoculum was added to obtain a final concentration of 5 × 10^5^ CFU/mL. Hereafter, the same remaining steps were done following the biofilm eradicative activity assay as detailed above.

The percentage of prevention or eradication was calculated using the following equation (2):

(2) $$\text{Eradication}\;\text{or}\;\text{Prevention}\;\%=\frac{O.D\;\text{positive}\;\text{control}-O.D\;\text{treated}\;\text{well}}{O.D\;\text{positive}\;\text{control}-O.D\;\text{negative}\;\text{control}}\;x\;100$$

### 2.12 Stereomicroscopy

The structure of *E. creticum* leaves and stems were observed using a microscope before and after the IVDV treatment. Pictures were taken using LEICA EZ4 HD stereomicroscope, and observations were performed using a magnification ranging between 8 × and 35 × with an ocular lens 10 × with scale.

### 2.13 RP-UHPLC-PDA-MS analysis

Reversed phase - ultra high-performance liquid chromatography - coupled to photodiode array detection and mass spectrometry (RP-UHPLC-PDA-MS) was used to analyze and identify the compounds in the IVDV-treated *E. creticum* leaves extract, achieved under the optimal conditions, using the equipment and method described before by Hammoud et al. [33].

### 2.14 Statistical analysis

Assays and measurements were accomplished in triplicate. Results were analyzed using ANOVA and expressed as average values ± standard deviations, and the statistical significance between experimental groups and control was determined by *p* values, with a significance level of 95% (p < 0.05). The extraction optimization process was statistically analyzed through STATGRAPHICS® Centurion XVII-X64 software, and the analysis of antibiofilm activities were done using GraphPad Prism® Software (Version 6.05, San Diego, CA, USA).

## 3 Results and discussion

### 3.1 Effect of water content, steam pressure and processing time on TPC yield and antiradical activity of the extract

**Fig. 2:**
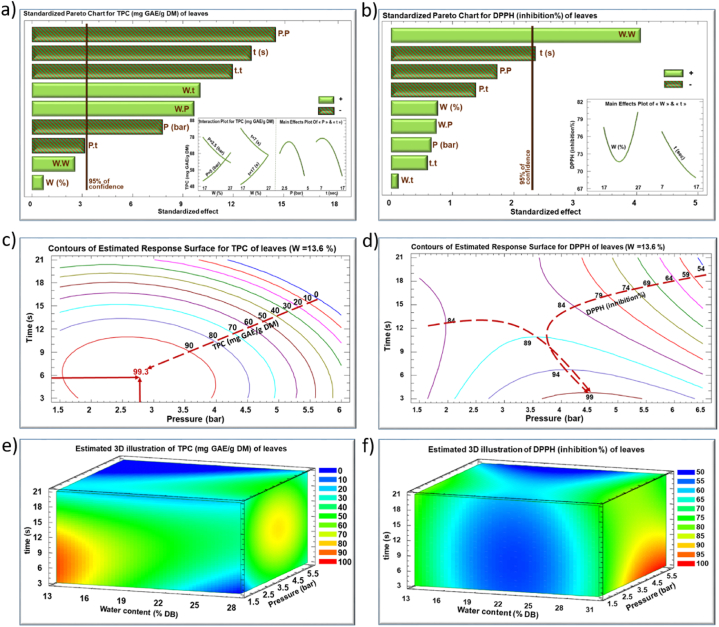
Standardized Pareto charts (a, b), their corresponding estimated response contours (c, d), and their corresponding estimated response surface mesh (e, f) of TPC and DPPH for IVDV treated *Eryngium creticum* leaves.

**Fig. 3:**
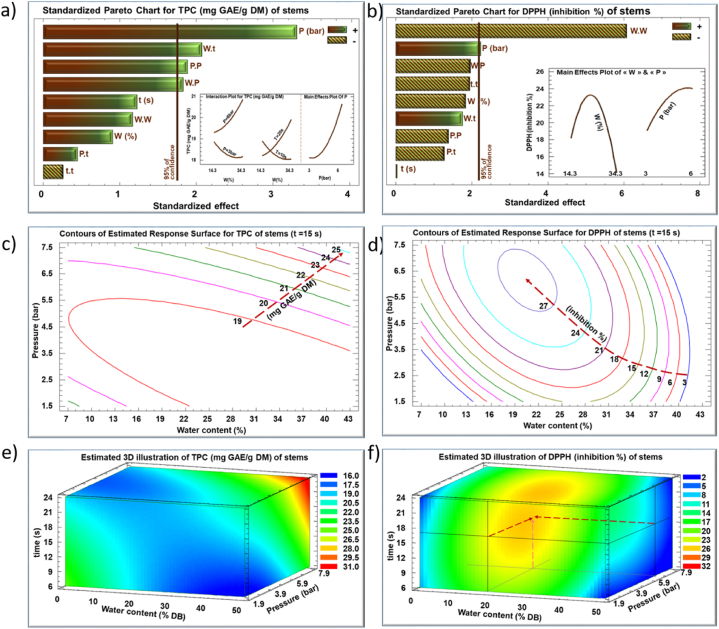
Standardized Pareto charts (a, b), their corresponding estimated response contours (c, d), and their corresponding estimated response surface mesh (e, f) of TPC and DPPH for IVDV treated *Eryngium creticum* stems.

**Fig. 4:**
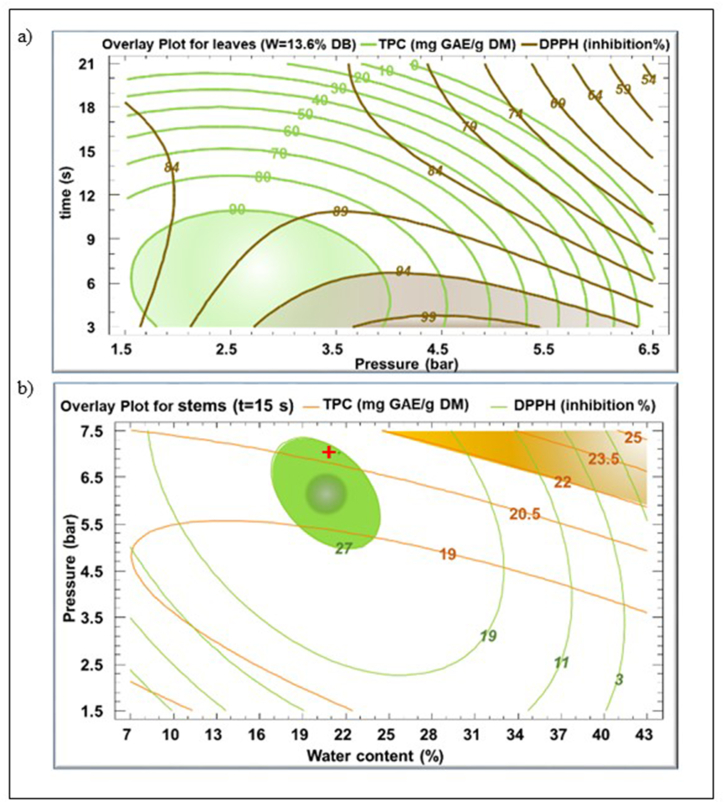
Contours plots and optimum values after multiple response analysis for TPC and DPPH inhibition percentage for *Eryngium creticum* leaves (a) and stems (b) extracts. The multiple optimum is indicated by a red cross.

The impact of IVDV processing parameters, water content (W) of the plant material, steam pressure (P), and processing time (t), on the recovery yield of polyphenols and their antiradical activity were quantified (Table 1, Fig. 2, Fig. 3). Multiple optimizations were suggested based on the second-degree empirical model (Fig. 4). Table 1 shows the TPC values and DPPH inhibition percentage values for *E. creticum* leaves and stems from the experimental design by RSM.

**Table 1:** Response parameters values (TPC and antiradical activity) of the IVDV treated *Eryngium creticum* leaves and stems.

Run		Leaves					Stems				
		Variable Levels Uncoded			**TPC (mg GAE/g DM)**	**DPPH inhibition percentage**	Variable Levels Uncoded			**TPC (mg GAE/g DM)**	**DPPH inhibition percentage**
		**Water content (%)**	**Steam pressure (bar)**	**Processing time (sec)**			**Water content (%)**	**Steam pressure (bar)**	**Processing time (sec)**		
**untreated**					60.71	74.7%	**Untreated**			13.02	3.7%
**Factorial design**	**1**	17	2.5	7	88.22	76.43	14.3	3	10	20.3	8.39
	**2**	27	2.5	7	43.17	80.19	34.3	3	10	16.64	4.30
	**3**	17	5	7	74.08	91.67	14.3	6	10	19.02	26.87
	**4**	27	5	7	62.14	90.68	34.3	6	10	19.82	7.26
	**5**	17	2.5	17	60.79	76.79	14.3	3	20	18.05	7.14
	**6**	27	2.5	17	50.13	70.25	34.3	3	20	19.15	9.01
	**7**	17	5	17	35.98	68.37	14.3	6	20	19.52	14.06
	**8**	27	5	17	58.28	79.75	34.3	6	20	20.69	8.79
**Star points**	**9**	13.6	3.75	12	59.07	85.75	7.5	4.5	15	18.41	5.67
	**10**	30.4	3.75	12	89.68	91.76	41.1	4.5	15	20.51	5.27
	**11**	22	1.64	12	58.68	63.09	24.3	1.97	15	18.23	19.07
	**12**	22	5.85	12	24.32	56.18	24.3	7	15	21.69	22.43
	**13**	22	3.75	4	62.65	74.58	24.3	4.5	7	17.77	16.43
	**14**	22	3.75	21	30.27	67.98	24.3	4.5	23	19.16	21.43
**Central repetition**	**15**	22	3.75	12	72.17	72.77	24.3	4.5	15	18.71	22.71
	**16**	22	3.75	12	70.93	72.01	24.3	4.5	15	18.48	22.00
	**17**	22	3.75	12	76.56	71.71	24.3	4.5	15	19.25	23.70
	**18**	22	3.75	12	73.1	71.41	24.3	4.5	15	17.67	23.23

The TPC values varied between 24.32 and 89.68 mg GAE/g DM, and between 16.64 and 21.69 mg GAE/g DM for the IVDV-treated leaves and stems extracts, respectively. The DPPH inhibition percentage varied between 56.18 and 91.76%, and 4.3 and 26.87% for the IVDV-treated leaves and stems extracts, respectively.

#### 3.1.1 Effect of processing parameters on *E*. *creticum* leaves extract

Fig. 2a and 2b show the Pareto charts representing the significant extraction parameters for TPC (Fig. 2a) and DPPH inhibition percentage (Fig. 2b) on the leaves of *E. creticum*. The effect of the IVDV processing parameters was determined according to these Pareto charts. The significance of parameters effect is indicated by bars crossing the vertical line with a confidence level of 95%. Hatched bars represent negative effects of the parameters, while others represent positive effects. Main effects of parameters and their interactions within the studied domains of variation are shown in the inserts.

The IVDV steam pressure (P) and processing time (t) had a significant negative linear effect on TPC from *E. creticum* leaves (Fig. 2a). Moreover, they showed significant negative quadratic effects (P.P and t.t), as confirmed in the insert (on the right) of Fig. 2 a. This quadratic negative effect is demonstrated by the parabolic pattern when the TPC value rises to reach a maximum and then decreases.

According to literature, saturated steam pressure, closely associated with the treatment temperature, appears to have the most critical effect on nearly all response parameters [34]. In such treatment, the high steam pressure drops towards vacuum and this leads to mechanical constraints within the product and the release of bioactive compounds from the cell matrix, thus increasing the extraction yield [20]. The sudden generation of a large amount of steam occurring during this phenomenon induces higher porosity in the material structure, resulting in an intensification of the diffusion of bioactive compounds during the extraction process, and a higher TPC yield. However, subjecting thin materials such as *E. creticum* leaves to treatment with “high-pressure” saturated steam followed by a sudden drop to the vacuum, may have two negative effects: the expulsion and loss of target molecules from the product (within the treatment chamber of IVDV) reducing the TPC yield in the pretreated leaves, and degradation and/or oxidation of these molecules due to the high treatment temperature. In the case of such thermally sensitive products, this is where we understand the importance of IVDV, which ensures a pressure rise of less than 1 s. Indeed, the optimum shown in Fig. 2 c is achieved with less than 6 s of treatment, whereas if the pressure rise requires an additional 10 s, such a product will have lost much of its beneficial compounds.

Similarly, the TPC yield is positively influenced by the processing time (t), up to 10 s of treatment. After this time, its effect became negative, and the quantity of TPC dropped dramatically. Longer treatment time at high temperature seems to affect negatively the stability of bioactive compounds, accounting for the negative linear effect of the IVDV processing time (t) on the DPPH inhibition percentage (Fig. 2b).

Nonetheless, a positive interaction between water content and steam pressure (W.P), and between water content and processing time (W.t) was observed for TPC yield. This is shown in the insert (on the left) of Fig. 2 a, representing the variation of the TPC yield as a function of water content W with varying steam pressure (P) or processing time (t) levels. The variation of TPC as a function of water content decreased when P was set at its low level (2.5 bars) but increased when P reached its high level (5 bars). At low pressure (relatively low temperature), the elasticity of the product is moderate. The increase in water content results in a higher generation of steam, and consequently to the breakage of the leaves and the expulsion of some of their TPC, leading to a reduction in their residual quantity. In contrast, the increase in water content at high pressure (high temperature) increases the elasticity of the product, preventing in this case their disintegrating and retaining the TPC in the product. Similarly, this may apply in the case of interaction between the water content and processing time.

Water content did not show a significant linear effect on TPC yield (Fig. 2a). Nevertheless, water content was proven to play a significant role in protecting the bioactive molecules to be extracted [19]. In this sense, this parameter showed a significant positive quadratic effect (W.W) (Fig. 2b) on the DPPH inhibition percentage, which could in this case be interpreted by the occurrence of two antagonistic phenomena. On one hand, the presence of water plays a protective role in preserving the biological activity of molecules of interest, such as polyphenols. On the other hand, an increase in water content contributes to the generation of a larger volume of steam during the pressure drop step. Consequently, this leads to an increase in the quantity of matter released out of the matrix, including polyphenols, ultimately mitigating the overall antioxidant activity of the extract. As a consequence, the measured DPPH will vary depending on which of these two phenomena prevails.

Fig. 2c and 2d represent the contours of estimated response surfaces of the *E. creticum* leaves extract. The contours illustrate the TPC and DPPH evolution as a function of steam pressure and processing time while maintaining the water content at 13.6% (optimal value suggested by the second-degree model).

The red discontinuous arrow in Fig. 2 c represents the TPC variation from 0 to 99.3 mg GAE/g DM. The overlapping of the two parabolic curves in the insert of Fig. 2 a leads to a TPC optimum of 99.3 mg GAE/g DM (tip of the arrow in Fig. 2c) by applying 2.8 bars as steam pressure for 5.5 s as processing time. This optimum is located in the center of the red ellipse. Likewise, the two discontinuous arrows in Fig. 2 d outline the pathways of DPPH increase. These two pathways converge toward an optimal region occurring at a time lower than 4 s and at a pressure ranging between 3.6 bars and 5.5 bars, for which 99% of the antiradical activity of the extract could be preserved.

Fig. 2e and 2f illustrate the variation of TPC and DPPH inhibition percentage, respectively, as a function of the three main processing parameters of IVDV (W, P, and t). The red and oranges zones indicate the maximum of TPC and DPPH inhibition percentage.

#### 3.1.2 Effect of processing parameters on *E. creticum* stems extract

Based on Fig. 3 a, only the IVDV steam pressure (P) showed significant linear and quadratic positive effects on the TPC yield of the extract from *E. creticum* stems. The high steam pressure induces a more intense expansion, accounting for its positive effect on the TPC yield. In fact, the pressure drop from a high level to vacuum is responsible for generating a high amount of steam inside the product, which, in turn, induces mechanical constraints resulting in the formation of alveoli (small cavities or pores) in the treated material [20]. Hence, the alveolated structure fosters solvent diffusion into the product and the extraction of biomolecules. Similar results were obtained in olive leaves [24] and chickpeas [35]. The IVDV-treated materials presented higher phenolic yield attributed to the rupturing of cellular structure, which enhances the accessibility of polyphenols, and release from the matrix.

The quadratic positive effect of steam pressure (P.P) is reflected by the existence of a latency phase in the beginning of the IVDV pretreatment (insert on the right in Fig. 3a).

While *E. creticum* stems needed a longer processing time for their expansion, a treatment of 4 s was sufficient in the case of the leaves due to their thin structure. In comparison to the leaves, *E. creticum* stems exhibit greater thickness, therefore requiring more intense processing conditions for their expansion, notably a wider pressure drop range. The subsequent expansion is clearly noticeable in this case, in contrast to the thinner leaves that may undergo structural disintegration if the conditions are intensified [19,36].

The interactions between water content and steam pressure (W.P) and water content and processing time (W.t) are shown in the insert (on the left) in Fig. 3 a. These two interactions positively affect the extraction yield by influencing the expansion strength and the release of cellular compounds.

According to the Pareto chart of Fig. 3 b, steam pressure (P) exhibited a linear positive effect on the DPPH inhibition percentage. As a matter of fact, the temperature rises while increasing the intensity of saturated steam pressure. Nonetheless, the elevated temperature may adversely affect the quality of the extracted bioactive compounds, due to thermal degradation. This explains the stabilization of DPPH inhibition percentage at steam pressures exceeding 5 bars.

The water content shows a significant negative quadratic effect on the antiradical activity of stems extract (Fig. 3b), in contrast to its impact on the *E. creticum* leaves extract. The higher water content resulted in greater mechanical constraints and intensified vapor formation, provoking the release and the loss of biomolecules from the matrix [37].

Fig. 3c and 3 d show the contours of estimated response surfaces for TPC and DPPH inhibition percentage, describing their evolution as a function of water content and steam pressure during 15 s. The maximum TPC yield of 25 mg GAE/g DM was reached while combining the highest steam pressure with the highest water content used (red dotted arrow) (Fig. 3c). However, the optimum of 27.5 DPPH inhibition percentage was reached at 6.3 bars and 21% as water content during 15 s (Fig. 3d). This result confirms a significant difference between leaves and stems, not only in terms of TPC and DPPH but also in the optimal processing conditions.

Fig. 3e and 3f show the TPC and DPPH inhibition percentage variation, respectively, as a function of the IVDV processing parameters. The zones in red and orange designate the maximum values of TPC and DPPH inhibition percentage. For TPC, the optimal zone was obtained by maximizing the three parameters (upper right red corner), whereas for the DPPH, the optimal zone is found inside the cube as indicated by the red dotted arrow.

#### 3.1.3 Multiple optimization and model validation

Simultaneous optimization of TPC and DPPH of the extracts was carried out in order to reveal the set of multiple optimum conditions. Fig. 4 shows the optimal conditions for *E. creticum* leaves extract (a) as a function of steam pressure and time where W is set at 13.6%, and for stems extract (b) as a function of water content and steam pressure during 15 s.

Fig. 4 a is an overlay of Fig. 2c and 2d. The green and the brown lines represent the variation of TPC and DPPH, respectively, and the colored areas indicate the optimum zones. An overlap was observed between TPC and DPPH optimum zones. The overlapped area allows to simultaneously reach a TPC yield higher than 90 mg GAE/g DM and a DPPH of approximately 99%.

Fig. 4b is an overlay of Fig. 3c and 3d. The orange and the green lines indicate the variation of TPC and DPPH, respectively. Conversely, the two optimum zones are set apart with no overlap observed. For this reason, a multiresponse optimization would be a compromise between the two optimums. A multiple optimum could be suggested with a TPC of 20.7 mg GAE/g DM and DPPH of 25.2% at 21.5% as water content and 7 bars during 15 s (red cross).

A good correlation between the model and the experimental results was obtained with a high R^2^. The fitted regression model, indicated by R^2^, reflects around 90% of the variability in the TPC response, for both *E. creticum* leaves and stems extracts. The experimental results were compared to the predicted values by the model. All results confirmed the predictability of the model for the extraction of bioactive compounds from IVDV-treated *E. creticum* leaves (green highlight in Table 2) and stems (yellow highlight in Table 2). The gray highlight in Table 2 shows the four models for TPC and DPPH for IVDV-treated *E. creticum* leaves and stems extracts, respectively.

**Table 2:** Multiple optimum conditions for IVDV-treated *Eryngium creticum* leaves and stems extracts. Comparison between predicted and experimental values, with the second-degree models suggested for TPC and DPPH of leaves and stems extracts.

Parameters	Multiple Optimization	
	Leaves	Stems
Water content W (%)	13.6	21.5
Steam pressure P (bar)	3.4	7
Processing time (sec)	4	15
TPC predicted values (mg GAE/g DM)	94.24	20.73
TPC experimental values (mg GAE/g DM)	89.07	20.06
DPPH predicted inhibition percentages (%)	95	27.2
DPPH experimental inhibition percentages (%)	93.51	27.54
TPC (leaves) = 185.28–12.065W + 19.40P + 0.145t + 0.07W^2^ + 1.32WP + 0.34 wt – 6.32P^2^ – 0.43 Pt – 0.32t^2^ TPC (stems) = 30.17–0.48W – 2.53P – 0.26t + 0.003W^2^ + 0.038WP + 0.013 wt + 0.21P^2^ + 0.018 Pt – 0.0025t^2^ DPPH (leaves) = 192.84–13.57W + 15.75P – 0.19t + 0.29W^2^ + 0.26WP + 0.01 wt – 1.96P^2^ – 0.49 Pt – 0.04t^2^ DPPH (stems) = – 74.10 + 3.29W + 16.31P + 2.57t – 0.07W^2^ – 0.19WP + 0.05 wt – 0.71P^2^ + 0.24 Pt – 0.09t^2^		

Comparing the experimental results shown in Table 2 with the results of raw materials shown in Table 1 and it is evident that IVDV treatment led to a substantial increase in the TPC yield in treated leaves and stems by 47% and 54%, respectively. Furthermore, there was a notable improvement in the DPPH inhibition percentage, increasing by 25% and 644% in IVDV-treated leaves and stems, respectively, when compared to their untreated counterparts.

The optimal conditions for IVDV treatment of leaves (water content 13.6%, steam pressure 3.4 bar, processing time 4 s) and stems (water content 21.5%, steam pressure 7 bar, and processing time 15 s) were used for subsequent analyses, including antioxidant, antibacterial, antibiofilm activities, as well as stereomicroscopy and RP-UHPLC-PDA-MS analysis.

### 3.2 Assessment of antioxidant activities of *E. creticum* extracts

The antioxidant capabilities of the *E. creticum* leaves and stems extracts were estimated using CUPRAC, ABTS and FRAP assays. The IVDV-treated *E. creticum* leaves extract showed higher antioxidant activities; 17.42 ± 0.13 TE mM, 728.45 ± 2.13 AA mM, and 2034.84 ± 1.18 Iron II μM, in comparison to the untreated leaves extract; 12.1 ± 0.38 TE mM, 428.75 ± 4.26 AA mM, and 1839.83 ± 5.89 Iron II μM for CUPRAC, ABTS and FRAP, respectively. Similarly, the IVDV-treated *E. creticum* stems extract showed elevated antioxidant activities, 5.08 ± 0.25 TE mM, 183.28 ± 10.65 AA mM, and 955.67 ± 14.14 Iron II μM, compared to the untreated stems extract, which showed values of 1.67 ± 0.08 TE mM, 79.37 ± 4.25 AA mM and 554.83 ± 3.53 Iron II μM for CUPRAC, ABTS and FRAP, respectively. Our findings are in agreement with prior studies that reported also higher antioxidant activity in leaf extracts compared to stem extracts in various plants, such as *Ferula gummosa* [38], *Rumex* L. [39], *Labisia paucifolia* [40], among others.

More importantly, extracts from IVDV-treated materials clearly exhibited enhanced antioxidant activities compared to untreated materials. These findings underscore that the use of IVDV as a pretreatment for plant materials prior to extraction yields higher TPC than that obtained from untreated plant materials. The central consideration, however, depends on the quality of the extracted compounds, ensuring they exhibit the desired valuable effects. Consequently, IVDV pretreatment has not only proven effective in intensifying TPC extraction yields from *E. creticum* leaves and stems but has also demonstrated efficacy in preserving the antioxidant activities of the extracts.

### 3.3 Antibacterial activity of *E. creticum* extracts

The antibacterial capacity of *E. creticum* leaves and stems extracts coupled to IVDV pretreatment was assessed, and the obtained MICs and MBCs are summarized in Table 3.

**Table 3:** MIC and MBC of IVDV treated *Eryngium creticum* leaves and stems extracts, and leaves untreated extract.

Plant extracts	MIC (mg/mL)				MBC (mg/mL)			
	*E. coli*	*P. aeruginosa*	*S. epidermidis*	*S. aureus*	*E. coli*	*P. aeruginosa*	*S. epidermidis*	*S. aureus*
**Leaves IVDV-treated**	50	100	50	100	100	>100^a^	100	>100^a^
**Stems IVDV-treated**	50	100	50	100	100	>100^a^	100	>100^a^
**Leaves untreated**	100	100	50	100	100	100	75	100

a The symbol (>) indicates the necessity of a higher concentration to check if there is an antibacterial effect.

No studies on the antibacterial capacity of *E. creticum* extracts after IVDV treatment had been reported previously. Our results show that both IVDV-treated *E. creticum* leaves and stems extracts exhibited comparable antibacterial capacities against the tested bacterial strains.

For Gram-negative bacteria, both IVDV-treated leaves and stems extracts were more effective against *E. coli*, with MIC and MBC values of 50 and 100 mg/mL, respectively, in comparison to *P. aeruginosa*, where the MIC was 100 mg/mL, and no detectable MBC was observed at the maximal concentration used.

Concerning Gram-positive bacteria, *S. epidermidis* exhibited greater sensitivity to both IVDV-treated leaves and stems extracts than *S. aureus.* The observed MICs were 50 and 100 mg/mL for IVDV-treated extracts from both leaves and stems against *S. epidermidis* and *S. aureus*, respectively. The MBC was 100 mg/mL against *S. epidermidis* but was not detectable against *S. aureus* at the maximal concentration used.

Although the MIC of IVDV-treated leaves extracts is higher than that of the untreated ones (50 and 100 mg/mL, respectively), the MBC registered against *E. coli* remained the same (100 mg/mL). As a result, the IVDV-treated leaves extract exhibited a higher antibacterial activity compared to the untreated sample against *E. coli*.

The IVDV-treated and untreated leaves extracts showed no difference in their MIC values against *P. aeruginosa* and *S. aureus* (100 mg/mL), and *S. epidermidis* (50 mg/mL), but their MBCs were not similar. No detectable MBCs for the IVDV-treated leaves extract compared to 100 mg/mL for the untreated ones against *P. aeruginosa* and *S. aureus* was observed, whereas it showed 75 and 100 mg/mL against *S. epidermidis* for the untreated leaves extract and IVDV-treated ones.

The antibacterial activity of leaves extracts was studied using different extraction techniques. A study on the antibacterial effect of leaves extracts using the infrared-assisted technique compared to water bath conventional technique was reported [27]. The *E. creticum* IR leaves extract revealed lower antibacterial activity against both *E. coli* and *S. epidermidis* than IVDV-treated extract, as their respective MIC values were equal to 75 and 50 mg/mL on both mentioned bacterial, respectively, despite having the same MBC (100 mg/mL) against the same aforementioned bacteria.

On the other hand, Makki et al. investigated the antibacterial activity of *E. creticum* extracts using a maceration technique for 8 h at room temperature, against *E. coli*, *S. aureus*, *S. epidermidis* CIP 444, *P. aeruginosa*, and *Enterococcus faecalis* [41]. They reported that both aqueous and ethanolic leaves extracts have an equal MIC against *S. epidermidis* CIP 444 (5 mg/mL). Although the same MIC and MBC were obtained against *S. epidermidis* CIP 444 for the aqueous extract, an MBC of 10 mg/mL was reported for the ethanolic extract. Additionally, no detectable MIC and MBC against *E. coli* on both aqueous and the ethanolic leaves extracts (value > 800 mg/mL) was measured. Furthermore, the ethanolic leaves extract exhibited an antibacterial activity with a respective MIC and MBC of 343 mg/mL against *P. aeruginosa*, and 294 mg/mL against *S. aureus*. The aqueous leaves extract exhibited an antibacterial activity with a respective MIC and MBC of 244 mg/mL against *P. aeruginosa*. No MBC was detectable against *S. aureus*, but an MIC equal to 354 mg/mL was reported for the aqueous leaves extract.

Based on our results, it was observed that IVDV treatment improves the antibacterial capacity of *E. creticum* extracts against *E. coli* compared to intact extracts, reducing the MICs values from 100 to 50 mg/mL. Nonetheless, the consistent MICs registered against *S. epidermidis*, *P. aeruginosa* and *S. aureus* for IVDV-treated and untreated extracts might explain the particularity of IVDV treatment in extracting specific bioactive against *E. coli*.

### 3.4 Antibiofilm activities of *E. creticum* extracts

The antibiofilm assay of IVDV-treated *E. creticum* leaves and stems extracts was performed on biofilms of *E. coli* and *S. epidermidis*, where biofilm eradication and prevention capabilities of the extracts were assessed. According to our knowledge, no studies on the antibiofilm activity of *E. creticum* leaves and stems extracts coupled to IVDV treatment were reported.

**Fig. 5:**
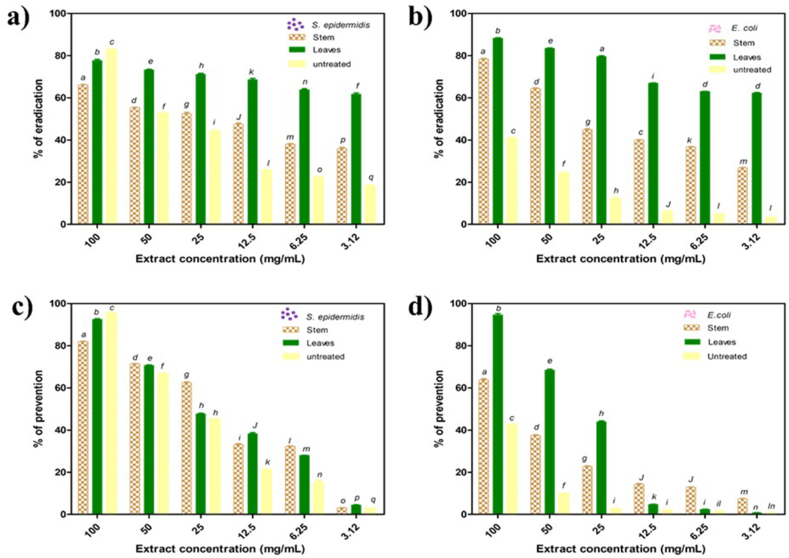
Biofilm eradication activity of *Eryngium creticum* extracts against *Staphylococcus epidermidis* (a) and *Escherichia coli* (b) and biofilm prevention activity of *E. creticum* extracts against *S. epidermidis* (c) and *E. coli* (d). Graphs of the different extracts showing the prevention or eradication percentage in function of extracts concentrations. Different letters indicate significant statistical difference.

Both IVDV-treated *E. creticum* leaves and stems extracts exhibited an eradicating antibiofilm capacities, but with variable patterns (Fig. 5). Our results indicated that IVDV-treated *E. creticum* leaves extract was more eradicating than stems extract. A concentration of 100 mg/mL of the IVDV-treated leaves extract was able to eradicate 88% and 77% of *E. coli* (Fig. 5b) and *S. epidermidis* (Fig. 5a) biofilms, respectively, while the IVDV-treated stems eradicated only 79% and 66% of the same biofilms extract. The IVDV-treated leaves extract was more eradicating than untreated leaves against *E. coli*, when the eradication percentage was 88% compared to 40% for the untreated one at the same concentration of 100 mg/mL (Fig. 5b). However, the untreated leaves extract was able to eradicate more the *S. epidermidis* biofilm (83%) than the IVDV-treated leaves extract (77%) (Fig. 5a).

Concerning biofilm prevention activity, IVDV-treated *E. creticum* leaves extract seems to prevent *E. coli* and *S. epidermidis* from forming biofilms more than IVDV-treated stems extract. The IVDV-treated leaves extract exhibited a stronger preventing antibiofilm activity than the untreated leaves against *E. coli* (Fig. 5d). The IVDV-treated leaves extracts showed a preventive percentage equal to 95% while the untreated ones succeed to prevent only 42% at the extract concentration of 100 mg/mL (Fig. 5d). The IVDV-treated leaves extract revealed a weaker preventing antibiofilm activity than the untreated one against *S. epidermidis* (Fig. 5c). The untreated leaves extract showed a preventive percentage equal to 95% while the IVDV-treated leaves extract was able to prevent only 93% (Fig. 5c). At the same concentration, the IVDV-treated leaves extract showed a higher preventive antibiofilm effect at 95% while only 62% for stems extract was obtained against *E. coli* (Fig. 5d). Although the preventive percentage of IVDV-treated leaves extract was 93% against *S. epidermidis*, the IVDV-treated stems extract was able to prevent only 82% (Fig. 5c). Thus, the IVDV-treated leaves extracts demonstrated a higher preventive antibiofilm activity than stems extracts. The antibiofilm prevention capacity of *E. creticum* leaves extracted by infrared-assisted technique compared to a solid-liquid conventional technique was reported [27]. The IVDV-treated leaves extract was able to prevent *E. coli* and *S. epidermidis* biofilms formation more than IR leaves extract. The concentration of 100 mg/mL of the IVDV-treated leaves extract was able to prevent *E. coli* and *S. epidermidis* from forming 95% and 93% of their biofilms, respectively. By contrast, the same concentration of the IR leaves extract was able to prevent *E. coli* and *S. epidermidis* from forming only 82% and 49% of their biofilms.

### 3.5 Stereomicroscopy observations

**Fig. 6:**
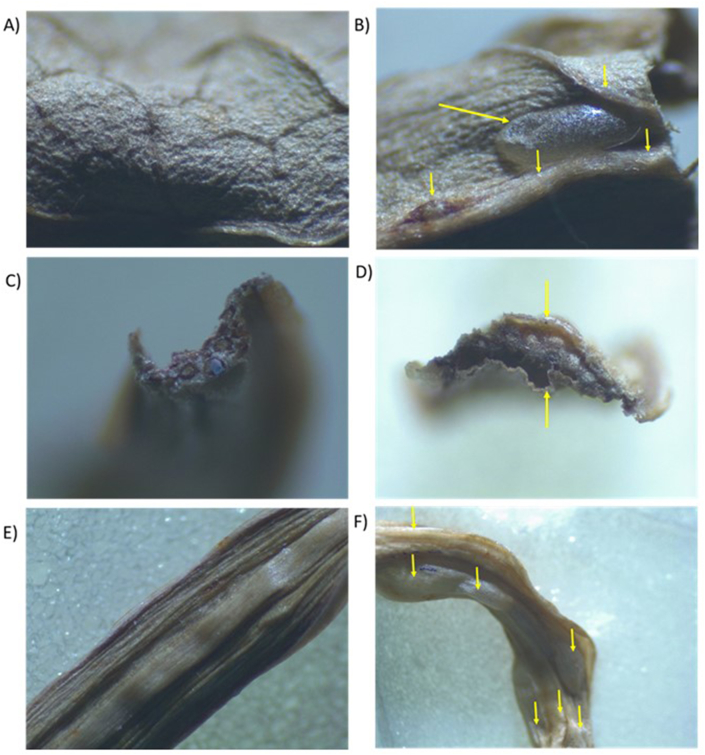
A) Structure of untreated *Eryngium creticum* leaves (350 × ), B) Alveolation of IVDV-treated *E. creticum* leaves (350 × ), C) Transversal section of untreated *E. creticum* stems (350 × ), D) Transversal section of IVDV-treated *E. creticum* stems (350 × ), E) Structure of untreated *E. creticum* stems (250 × ), F) Alveolation of IVDV-treated *E. creticum* stems (125 × ). Arrows indicate the formation of pockets, bubbles, or alveoli, at the surface of the product.

Stereomicroscopy was used to observe the structural modifications that may have occurred of *E. creticum* leaves and stems following the IVDV treatment. Fig. 6 shows the pictures of the external structure of *E. creticum* leaves and stems before (6.A and 6.E) and after (6.B and 6.F) the IVDV pretreatment. Arrows indicate the formation of pockets, bubbles, or alveoli, at the surface of the product. This alveolation contributes in enhancing the diffusivity of the solvent inside the plant framework, which explains the improvement of the extraction process of IVDV-pretreated parts. Fig. 6C and 6.D show the pictures of the transversal sections of *E. creticum* stems without and with the IVDV pretreatment, respectively. While Fig. 6C presents compacted material, Fig. 6D reveals a more voluminous, or expanded, material. All these Figs. (6B, 6C, 6D and 6F) confirm that the IVDV process affects the structure of the plant matrix, in a way that it promotes both the diffusivity of the solvent and the extractability of the biomolecules.

### 3.6 Analysis of bioactive compounds by RP-UHPLC-PDA-MS

**Fig. 7:**
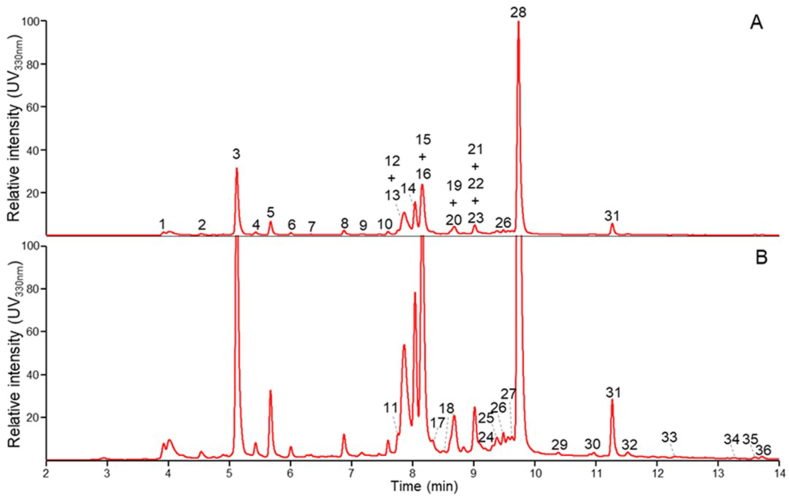
RP-UHPLC-UV330 nm profiles of *Eryngium creticum* IVDV-treated leaves extract at full (A) and 5× zoomed intensity (B). Numbers correspond to tentatively identified compounds in Table 4.

Numerous phenolic compounds in *E. creticum* IVDV-treated leaves extract (obtained under the optimal IVDV conditions) were analyzed by RP-UHPLC-PDA-MS. The retention time, UV absorbance maximum and fragmentation were compared to the literature [42]. Different phenolic compounds were detected and are shown in Fig. 7.

**Table 4:** Phenolic compounds tentatively identified in *Eryngium creticum* IVDV-treated leaves extract.

No.	R_t_ UV (min)	*λ*_max_ (nm)	R_t_ MS (min)	[M − H]^-^ (*m/z*)	MS^2^ fragments^a^	Tentative annotation^b^
1	3.93	322	4.02	353	191, 179, 135	1-*O*-Caffeyolquinic acid
2	4.55	314	4.62	343	179, 161, 135	Caffeic acid glucoside der.
3	5.12	326	5.21	353	191, 179, 135	3-*O*-Caffeyolquinic acid
4	5.43	326	5.52	353	191, 179, 135	4-*O*-Caffeyolquinic acid
5	5.68	322	5.77	179	135	Caffeic acid
6	6.01	322	6.11	253	135, 161, 179	Caffeic acid der.
7	6.28	318	6.37	337	191, 163	*p*-Coumaroylquinic acid
8	6.88	326	6.97	367	191, 173, 193	5-Feruloylquinic acid
9	7.17	326	7.27	431	385, 179, 161	?
10	7.60	346	7.69	593	447, 301	?
11	7.76	350	7.85	609	301, 343, 271	Quercetin rhamnosylgalactoside
12	7.87	302	7.96	487	355, 443, 337, 293	?
13	7.87	n.d.	7.97	609	301, 343, 271	Quercetin rutinoside
14	8.04	354	8.13	463	301, 343, 179	Quercetin galactoside
15	8.15	n.d.	8.24	477	301	Quercetin glucuronide
16	8.16	354	8.26	463	301, 343, 179	Quercetin glucoside
17	8.33	338	8.42	593	285, 327, 255	Luteolin rutinoside
18	8.63	n.d.	8.72	607	463, 505, 545, 301	Diosmetin rutinoside
19	8.68	350	8.77	505	301, 463	Quercetin acetylglycoside
20	8.68	n.d.	8.80	447	284, 285, 327, 255	Luteolin galactoside
21	9.02	330	9.10	447	284, 285, 327, 255	Luteolin glucoside
22	9.02	330	9.11	461	285, 175, 299	Diosmetin glucoside
23	9.02	n.d.	9.13	477	314, 357, 301, 449	Isorhamnetin galactoside
24	9.20	n.d.	9.29	477	314, 357, 285, 301	Isorhamnetin glucoside
25	9.32	n.d.	9.41	491	315, 301	Isorhamnetin glucuronide
26	9.49	342	9.59	635	446, 489, 301, 593	Luteolin acetylrutinoside
27	9.57	326	9.66	515	353, 299, 202	Dicaffeoyl quinic acid
28	9.74	330	9.82	359	197, 179, 197	Rosmarinic acid
29	10.39	330	10.48	619	431, 473, 285	Luteolin rutinoside der.
30	10.95	326	11.04	343	161, 181, 135	Isorinic acid
31	11.27	330	11.37	373	179, 135, 161	Rosmarinic acid methyl ester
32	11.53	362	11.62	301	179, 151, 273, 257	Quercetin
33	12.30	322	12.39	677	489, 301, 635	Luteolin diacetylrutinoside
34	13.25	334	13.34	285	285, 151, 257	Luteolin
35	13.61	322	13.70	327	229, 171, 291	?
36	13.72	324	13.81	313	161, 269	?

λ_max_ compared to literature; n.d. not determined; ?: compound could not be tentatively annotated.

a fragments arranged in decreasing intensity order.

b based on fragmentation, retention time.

The numbers in Fig. 7 (7.A and 7.B) refer to the compounds shown in Table 4. These compounds are tentatively identified. Based on UV intensity, caffeic acid derivatives and flavone and flavonol glucosides were pinpointed as the most abundant compounds in *E. creticum* IVDV-treated leaves extract, with 3-O-caffeyolquinic acid (Fig. 7A) and rosmarinic acid (Fig. 7B) standing out in terms of abundance.

As mentioned, the medicinal properties of plants have previously been related to their phytochemicals profile. Caffeoylquinic acid and its derivatives have a variety of bioactivities. These compounds are renowned as potential antioxidants [43], additionally to their important antitumor, antimicrobial, anti-inflammatory activities, and others [44]. 3-O-Caffeoylquinic acid, as a key active compound identified in the *E. creticum* IVDV-treated leaves extract could therefore underly the high antioxidant capacity found. Likewise, caffeic acid is recognized for its biological abilities including powerful antioxidant activity, anti-inflammatory, anticancer, antiviral capabilities, and others [45]. Several studies have documented the occurrence of caffeic acid in plant species known for their anti-snake venom properties [46]. Consequently, the presence of caffeic acid in the leaf extract of *E. creticum* may contribute to its antidotal effects and justify its inclusion in pharmacopeias as an alternative treatment for snakebites [2]. In addition, quercetin, and its derivatives, display a widespread range of biological activities including antibacterial, antiviral, anti-inflammatory, antioxidant, and therapeutic, leading to cosmetic, pharmaceutical, and food industries applications [47].

Numerous studies have shown the biological properties of rosmarinic acid; it is mainly renowned for its potent antioxidant and anti-inflammatory properties [48,49]. Rosmarinic acid has also been used in pharmacopeia for relieving pain and headaches. Hence, the biological capabilities of *E. creticum* extracts could likely be attributed to the identified phenolic compounds. *E. creticum* extracts could be a functional ingredient for several food, medicine, and pharmaceutical products.

## 4 Conclusions

Our results emphasize the significance of *E. creticum* extracts, highlighting their antioxidant, antibacterial, and antibiofilm capacities. The extracts from leaves demonstrated higher TPC yield, higher antiradical and antioxidant activities, and superior prevention and eradication of biofilm capacities than those from stems. The phytochemical profile of *E. creticum* extracts revealed large number of bioactive compounds potentially essential for their biological activities.

Furthermore, the effectiveness of the IVDV process as a pretreatment on *E. creticum* extracts was demonstrated in this study. The extremely rapid pressure increases in an IVDV cycle, from vacuum to 12 bars in less than 1 s, is of high importance, especially when optimal thermal treatment time does not require more than few seconds. In contrast, if the pressure rise takes 5–10 s (and even longer in the case of other processes), it can severely compromise the product quality.

The IVDV process enables precisely timed treatment, ensuring a desired treatment. It can be introduced as an innovative pretreatment applied before the extraction step in view of enhancing the recovery of targeted bioactive compounds from medicinal plants, while protecting their biological activities compared to conventional methods. IVDV has evolved to become versatile tool for extracting valuable bioactive constituents from plants, underscoring its potential contribution to the field of plant-based biomolecules research and application.

## Funding

This project has been jointly funded with the support of the 10.13039/501100005993National Council for Scientific Research of Lebanon 10.13039/501100007175CNRS-L and Saint-Joseph University of Beirut (10.13039/501100004338USJ). The project also received funding from the EU BRISK II project (10.13039/501100007601Horizon 2020, Grant agreement No 731101) which was co-financed by the WUR Knowledge Base Program (KB34-009-007).

## Data availability statement

Data will be made available on request.

## CRediT authorship contribution statement

**Mariam Hammoud:** Writing – review & editing, Writing – original draft, Validation, Resources, Methodology, Investigation, Data curation. **Espérance Debs:** Writing – review & editing, Writing – original draft, Validation, Resources, Investigation, Formal analysis. **Lambertus A.M. van den Broek:** Writing – review & editing, Validation, Resources, Investigation, Funding acquisition. **Hiba N. Rajha:** Writing – review & editing, Validation, Supervision, Resources. **Carl Safi:** Writing – review & editing, Validation, Resources, Funding acquisition. **Gijs van Erven:** Writing – review & editing, Validation, Resources. **Richard G. Maroun:** Writing – review & editing, Validation, Supervision, Resources, Methodology, Funding acquisition, Formal analysis, Data curation, Conceptualization. **Ali Chokr:** Writing – review & editing, Validation, Supervision, Resources, Methodology, Formal analysis, Data curation, Conceptualization. **Hassan Rammal:** Validation, Supervision, Resources, Methodology, Investigation, Formal analysis, Data curation, Conceptualization. **Nicolas Louka:** Writing – review & editing, Validation, Supervision, Resources, Project administration, Methodology, Funding acquisition, Formal analysis, Data curation.

## Declaration of competing interest

The authors declare that they have no known competing financial interests or personal relationships that could have appeared to influence the work reported in this paper.
